# Risks of organ failures and deaths associated with young-onset dementia after hospitalizations for motor vehicle crash injuries: a nationwide population-based retrospective cohort study

**DOI:** 10.1038/s41598-023-30868-6

**Published:** 2023-03-13

**Authors:** Chien-Hui Liu, Jiun-Yi Wang, Kun-Chia Chang, Ming-Chung Ko, Pei-Chen Lee, Chih-Ching Liu

**Affiliations:** 1 Institute of Biomedical Informatics, National Yang Ming Chiao Tung University, Hsinchu, Taiwan; 2 Division of Emergency Medical Service, New Taipei City Fire Department, New Taipei City, Taiwan; 3 Department of Healthcare Administration, College of Medical and Health Science, Asia University, Taichung, Taiwan; 4 Department of Medical Research, China Medical University Hospital, China Medical University, Taichung, Taiwan; 5 Jianan Psychiatric Center, Ministry of Health and Welfare, Tainan, Taiwan; 6 Department of Psychiatry, National Cheng Kung University Hospital, College of Medicine, National Cheng Kung University, Tainan, Taiwan; 7 Department of Surgery, Zhong-Xing Branch, Taipei City Hospital, Taipei, Taiwan; 8 Department of Public Health, College of Medicine, National Cheng Kung University, Tainan, Taiwan

**Keywords:** Dementia, Epidemiology, Health services

## Abstract

Patients with dementia are at increased risks of adverse consequences associated with motor vehicle crash injury (MVCI). However, studies of the association for patients with young-onset dementia (YOD) are limited. Therefore, we aim to investigate whether YOD was associated with adverse outcomes after hospitalization for MVCI. In this retrospective cohort study, we identified 2052 MVCI patients with YOD (aged 40–64 years) between 2006 and 2015 and included 10 260 matched MVCI patients without YOD (matching ratio: 1:5) from Taiwan’s National Health Insurance Research Database and the Taiwan Police-Reported Traffic Accident Registry. We evaluated the intensive care unit (ICU) admission, organ failure, in-hospital and 30-day mortalities, length of hospital stay, and hospital costs. Compared with participants without dementia, patients with YOD had higher rates of ICU admission (34.31% vs. 20.89%) and respiratory failure (6.04% vs. 2.94%), with a covariate-adjusted odds ratio of 1.50 (95% CI 1.33–1.70) and 1.63 (95% CI 1.24–2.13), respectively. The patients also exhibited higher in-hospital mortality (4.73% vs. 3.12%) and 30-day mortality (5.12% vs. 3.34%) than their non-YOD counterparts, but the risk ratio was not significant after adjusting for transport mode. Moreover, the log means of hospital stay and cost were higher among patients with YOD (0.09 days; 95% CI 0.04–0.14 and NT$0.17; 95% CI 0.11–0.23, respectively). This cohort study determined that YOD may be adversely associated with hospital outcomes among MVCI patients. However, the association between YOD and mortality risk may depend on transport mode.

## Introduction

Road traffic accidents (RTAs) are the eighth leading cause of death among all ages of the global population and are associated with considerable economic loss^1^. Motor vehicle crash injuries (MVCIs) account for more than half of all worldwide road traffic deaths^1^. The prognosis of victims of MVCIs depends on demographics, the severity of the mechanical traumatic force, and the comorbidities of these victims^1–5^.

Dementia affects approximately 50 million people worldwide^6^. According to previous studies, having dementia is associated with a risk of injuries from motor vehicle crashes, with the association spanning accidents involving cars, motorcycles, bicycles, and pedestrians^7–11^. Moreover, having dementia is associated with a greater risk of hospitalization, poor health outcomes, and an increased mortality rate^12,13^. People with dementia experience approximately 2 times higher medical costs and a twofold longer length of hospital stay (LOS) than a healthy comparison group^14,15^.

Despite the aforementioned results, it remains unknown whether dementia may still contribute additional risk to hospitalized patients with MVCI who may have experienced adverse outcomes. Given that studies have reported road traffic mortality to be considerably higher in younger adults than in older adults^1,4,5^ and that young-onset dementia (YOD) may have a more malignant course than older-onset dementia^16^, we focused on younger adults and conducted a nationwide, population-based cohort study to explore whether YOD is associated with a higher risk of intensive care unit (ICU) admission, organ failure, or death, longer hospital LOS, or greater hospital cost among hospitalized MVCI patients.

## Methods

### Data source

Data were obtained from two national registers: Taiwan’s National Health Insurance Research Database (NHIRD) and Taiwan’s Police-Reported Traffic Accident Registry (PTAR).

The NHIRD includes all outpatient and inpatient medical claims data of Taiwan’s population covered by National Health Insurance (NHI); the NHI program has been implemented in Taiwan since 1995 and has covered more than 99% of Taiwan’s residents since 2014^17^. To ensure the accuracy of the claims data in the NHIRD, the National Health Insurance Administration (NHIA) performs expert reviews on a random sample of every 50–100 ambulatory and inpatient claims quarterly^18^, and false reports of diagnoses incur a severe penalty from the NHIA. Therefore, the information obtained from the NHIRD is considered to be complete and accurate.

Information on RTAs, vehicles, and victims was obtained from Taiwan’s PTAR, which is released by the Taiwan National Police Agency. In Taiwan, vehicle accident reports are investigated and recorded by qualified and experienced police officers who handle road traffic crashes based on the “Regulation Governing Road Traffic Accidents”^19^. Thus, the crash reports including MVCIs in the PTAR are considered reliable. Other details of the PTAR have been provided in previous studies^11,20^. Written informed consent from the subjects was waived as all data were de-identified. Additionally, waiver for informed consent is approved by the Institutional Review Board of Jen-Ai Hospital (JA-109–83). All methods were carried out in accordance with relevant guidelines and regulations. All experimental protocols were approved by a named institutional and/or licensing committee/s.

### Study design and sampled participants

We conducted a nationwide, population-based, retrospective cohort study. All people aged 40 to 64 years who were hospitalized with an admission for an MVCI between 2006 and 2015 were enrolled as study participants. To ensure that the vehicle crash definition was reliable and consistent, the participants were considered hospitalized for an MVCI if (1) they had a diagnosis of MVCI (*International Classification of Diseases, 9th Revision, Clinical Modification* [*ICD-9-CM*] codes E810-E829) and (2) the vehicle crashes were also recorded in the PTAR^11^. Additionally, when people had more than one hospitalization for an MVCI, only the first hospitalization between 2006 and 2015 was included in the analysis. Participants having diagnostic codes of dementia (*ICD-9-CM* codes of 290, 291, 294, 331, 046.1) before or at the time of their MVCI-related hospitalization were considered the dementia group^21–23^, and those without dementia were classified as the nondementia group. To avoid accidental inclusion of miscoded patients, in the dementia group, patients were excluded if they had at least two these dementia-related diagnostic codes less than 90 days apart^21–23^. The nondementia cohort randomly selected from the nondementia group was frequency-matched to the dementia group by age (each 5-year span), gender, and index year at a 1:5 ratio. The index date was the first date of hospitalization for an MVCI for all study participants.

### Covariates

The salary-based insurance premium was adjusted to account for possible individual socioeconomic differences in the prognosis of hospitalization for an MVCI^24^. The urbanization level of traffic crash location (urban, suburban, or rural) and accreditation of medical institutions (medical center [> 500 beds], regional hospital [250–500 beds], or district hospital [20–249 beds]) were regarded as covariates to minimize the potential influence of differential accessibility and availability of medical resources. The urbanization level was measured according to the method developed and defined by Liu et al^25^. Comorbidities contributing to the Charlson comorbidity index (CCI) were identified by utilizing the *ICD-9-CM* coding developed by Deyo for use on administrative databases^26^. We excluded dementia itself in the CCI to avoid collinearity between dementia and the CCI^27^. The transportation mode (ie, car, motorcycle, bicycle, pedestrian, other) that resulted in a patient’s MVCI-related hospitalization was assessed according to the PTAR record. The types of injury (Supplementary Table 1) and body parts injured (Supplementary Table 2) in the crash were determined on the basis of the *ICD-9-CM* codes in the NHIRD. The TMPM (Trauma Mortality Prediction Model) was used to evaluate the individual trauma injury severity among inpatients. Calculation of the TMPM relied on the severity of body regions based on the ICD-9-CM codes in the NHIRD. Using the open-access R statistical software (package: ICDPIC-R), corresponding severity scores (range, 0–1) which showed direct association with the likelihood of mortality^28,29^ were then calculated.

### Outcome measures

Hospital outcomes in the present study were ICU admission, organ failure, in-hospital and 30-day mortalities, LOS, and hospital costs. The presence of acute organ dysfunction was identified after the first hospitalization for an MVCI and assessed according to the *ICD-9-CM* codes (Supplementary Table 3). Thirty-day mortality, in a process recommended by the World Health Organization^30^, was calculated by dividing the number of deaths by the total number of study participants within 30 days of the first hospitalization for an MVCI. LOS was defined as the difference in days between the admission date and final discharge date of the first hospitalization for an MVCI. Hospital cost was the total of any medical expenses incurred during the hospital stay for an MVCI.

### Statistical analysis

Differences in the distribution of categorical variables between MVCI patients with and without dementia were compared using the chi-squared test. Considering that participants from the same medical institution could have a higher correlation than those from different medical institutions, Generalized Estimating Equations (GEE) was used to take into account potential clustering effects^31^. Logistic regression model with GEE were used to estimate the adjusted odds ratio (AOR) of ICU admission, acute organ failure, and mortality, with the 95% confidence interval (CI) between patients with and without dementia. To better understand the association of dementia with mortality, six sets of covariates were applied in the following models: Model 0 used the null model (i.e., no covariates were added in the Model 0); Model 1 included patient characteristics (gender, age, salary-based insurance premium, admission year, CCI, and urbanization) and the accreditation of medical institutions; Model 2 included patient characteristics, the accreditation of medical institutions, and LOS; Model 3 included the number of body parts injured, surgical operations, and covariates in Model 2; and Model 4 was adjusted for transport mode and the covariates in Model 3; and Model 5 was adjusted for TMPM and the covariates in Model 4.

LOS and hospital cost were ascertained by performing log-transformation because of the right-skewed distribution. Additionally, separate univariable and multivariable linear regression models with analysis using GEE^31^ were applied to compare the difference with their 95% CI in LOS and hospital cost between MVCI patients with and without dementia. The covariates in these models included gender, age, salary-based insurance premium, admission year, CCI, urbanization, medical institution accreditation, number of body parts injured, TMPM, and transport mode. A *p*< 0.05 was considered significant. All statistical analyses were performed using SAS, version 9.4 (SAS Institute Inc., Cary, NC).

## Results

### Demographic and clinical characteristics of study participants

Table 1 presents the demographic and clinical characteristics of the two study groups. A total of 12 312 MVCI patients (aged 40–64 years) were enrolled in this study. Of them, 2052 had dementia. The gender proportion and age distributions exhibited no difference between the cohorts with and without dementia. Compared with the cohort without dementia, the dementia cohort had a lower insurance premium and were less likely to be treated in medical centers but had a higher prevalence of comorbidities and a higher ratio of traffic crashes in rural areas. The cohort with dementia had a greater number of body parts injured, a higher TMPM, and included more cyclists, pedestrians, and other types of vehicle users. Moreover, brain, chest, abdomen, skin, and other body part injuries were more frequent in the cohort with dementia, but the percentage of patients who underwent a surgical operation was similar in both groups.

**Table 1:** Demographic and clinical characteristics of study participants for 2006 to 2015.

Variables^†^	MVCI with dementia		MVCI without dementia		p*
	n	%	n	%	
Total	2052	100.0	10,260	100.0	
Gender					
Men	1512	73.7	7560	73.7	> 0.99
Female	540	26.3	2700	26.3	
Age (years)					> 0.99
40–44	442	21.5	2210	21.5	
45–49	449	21.9	2245	21.9	
50–54	402	19.6	2010	19.6	
55–59	342	16.7	1710	16.7	
60–64	417	20.3	2085	20.3	
Mean ± SD	51.6 ± 7.3		51.6 ± 7.3		
Salary-based insurance premium (NTD)					<0.001
<1st Tertile^‡^	1203	58.9	2916	28.6	
≥ 1st–2nd Tertile	475	23.2	3510	34.5	
≥ 2nd Tertile	365	17.9	3763	36.9	
Mean ± SD	17,424.9 ± 14,108.4		25,899.4 ± 20,569.3		
CCI					<0.001
0	1171	57.0	8494	82.8	
1	484	23.6	1048	10.2	
≥ 2	397	19.4	718	7.0	
Mean ± SD	0.87 ± 1.43		0.31 ± 0.89		
Comorbidities^§^					
Stroke	253	12.3	312	3.0	<0.001
Diabetes mellitus	399	19.4	1591	15.5	<0.001
Hypertension	609	29.7	2382	23.2	<0.001
COPD	139	6.8	399	3.9	<0.001
CKD	91	4.4	323	3.1	0.003
Urbanization					<0.001
Rural	384	19.0	1497	14.8	
Suburban	906	45.0	4281	42.4	
Urban	725	36.0	4314	42.8	
Accreditation of medical institution					0.008
Medical center	441	21.6	2525	24.8	
Regional hospital	1133	55.6	5475	53.8	
District hospital	465	22.8	2175	21.4	
Transport mode					<0.001
Driver of motor vehicle	126	6.14	896	8.73	
Motorcyclist	1396	68.03	7855	76.56	
Pedal cyclist	173	8.43	477	4.65	
Pedestrian	259	12.62	592	5.77	
Else	98	4.78	440	4.29	
Body part injured					
Brain	929	45.27	2935	28.61	<0.001
Chest	202	9.84	844	8.23	0.016
Limbs	1547	75.39	7945	77.44	0.044
Abdomen	95	4.63	350	3.41	0.007
Skin	1188	57.89	5276	51.42	<0.001
Others	716	34.89	2041	19.89	<0.001
Number of body parts injured					<0.001
1	525	25.58	3796	37.00	
2	717	34.94	4075	39.72	
> 2	810	39.47	2389	23.28	
MVC injury severity (TMPM probability of death)					<0.001
<Median^#^	391	19.05	2881	28.08	
≥ Median	1661	80.95	7379	71.92	
Mean ± SD	0.062 ± 0.104		0.040 ± 0.082		
Surgical operation	1027	50.05	5146	50.16	0.929

*MVCI* motor vehicle crash injury, *SD* Standard deviation, *NTD* New Taiwan Dollars, *CCI* Charlson comorbidity index, *COPD* Chronic obstructive pulmonary disease, *CKD* Chronic kidney disease, *MVC* motor vehicle crash, *TMPM* Trauma Mortality Prediction Model.

^†^Inconsistency between total population and population summed for an individual variable was due to missing information.

^‡^1st tertile = 19,200; 2nd tertile = 25,200.

^§^Baseline comorbidities diagnosed within 1 year before the index date.

^#^Median = 0.013.

*Based on χ^2^ test for category variables.

### Associations between dementia and hospitalization outcomes

Table 2 presents the association of dementia with the risk of ICU admission and organ failure in MVCI patients. After adjustment for baseline covariates, dementia was associated with an increased risk of ICU admission (odds ratio [OR]: 1.50; 95% confidence interval (CI), 1.33–1.70) and respiratory failure (OR: 1.63; 95% CI, 1.24–2.13). However, the risks of cardiovascular, hepatic, hematologic, and other failures (ie, renal, neurologic, and metabolic organ failure) exhibited no significant difference between the two groups after adjusting for baseline covariates.

**Table 2:** Association of dementia with the risk of ICU admission and organ failure in all patients with MVCI.

Variables	MVCI with dementia^§^	MVCI without dementia^§^	Crude OR (95% CI)	*P*	AOR^†^ (95% CI)	*P*
	(n = 2052)	(n = 10,260)				
ICU admission	34.31	20.89	2.01(1.81–2.23)	<0.001	1.50(1.33–1.70)	<0.001
Organ failure						
Any	6.97	3.31	2.22(1.80–2.73)	<0.001	1.60(1.25–2.06)	<0.001
Respiratory	6.04	2.94	2.17(1.74–2.71)	<0.001	1.63(1.24–2.13)	<0.001
Cardiovascular	0.58	0.23	2.73(1.35–5.53)	0.01	1.56(0.71–3.45)	0.27
Hepatic	0.34	0.19	1.66(0.66–4.20)	0.28	1.06(0.36–3.12)	0.92
Hematologic	0.49	0.13	3.75(1.58–8.92)	0.01	2.24(0.81–6.19)	0.12
Other^‡^	0.29	0.13	2.31(0.88–6.70)	0.09	1.22(0.41–3.67)	0.72

*MVCI* motor vehicle crash injury, *ICU* intensive care unit, *OR* odds ratio, *AOR* Adjusted odds ratio.

^†^Based on logistic regression with generalized estimating equations models with adjustment for dementia, gender, age, salary-based insurance premium, year of admission, CCI, urbanization, accreditation of medical institution, number of body parts injured, TMPM, and transport mode.

^‡^Due to the fact that renal, neurologic, and metabolic organ failure were seen in a relatively small number of patients, they were regrouped to one group, namely “others,” to obtain more reliable estimates.

^§^Per 100.

Table 3 compares in-hospital and 30-day mortalities between the two study groups. Compared with the cohort without dementia, the cohort with dementia had higher rates of in-hospital mortality (4.73% vs. 3.12%) and 30-day mortality (5.12% vs. 3.34%). After adjustment for patient demographics, salary-based insurance premium, admission year, CCI, urbanization, and medical institution accreditation in Model 1, the cohort with dementia had 34% (95% CI, 1.04–1.74) and 28% (95% CI, 1.01–1.65) higher risks of in-hospital and 30-day mortalities, respectively, than the cohort without dementia. The risk of these adverse outcomes was markedly increased after further adjusting for LOS in Model 2 and then slightly decreased, but still significant after further adjusting for the number of body parts injured and surgical operations in Model 3. However, the cohort with dementia had no significantly increased risk of in-hospital mortality (OR: 1.26, 95% CI, 0.96–1.65) or 30-day mortality (OR: 1.22, 95% CI, 0.94–1.59) after further adjusting for transport mode in Model 4. The risks of these adverse outcomes were similar to Model 4 after further adjusting for TMPM in Model 5.

**Table 3:** Association between dementia and in-hospital and 30-day mortalities in patients with MVCI.

Outcomes	MVCI with dementia	MVCI without dementia	Model 0		Model 1		Model 2		Model 3		Model 4		Model 5	
	%	%	Crude OR (95%CI)	*P*	AOR^†^ (95%CI)	*P*	AOR^†^ (95%CI)	*P*	AOR^†^ (95%CI)	*P*	AOR^†^ (95%CI)	*P*	AOR^†^ (95%CI)	*P*
In-hospital mortality	4.73	3.12	1.68 (1.32–2.13)	0.01	1.34 (1.04–1.74)	0.03	1.48 (1.14–1.92)	0.01	1.41 (1.08–1.83)	0.01	1.26 (0.96–1.65)	0.09	1.21 (0.93–1.59)	0.16
30-day mortality	5.12	3.34	1.69 (1.35–2.13)	0.01	1.28 (1.01–1.65)	0.04	1.46 (1.13–1.88)	0.01	1.36 (1.05–1.77)	0.02	1.22 (0.94–1.59)	0.14	1.18 (0.91–1.55)	0.21

*MVCI* motor vehicle crash injury, *OR* odds ratio, *AOR* adjusted odds ratio, *CI* confidence interval.

^†^Model 1 was adjusted for dementia, gender, age, year of admission, salary-based insurance premium, CCI, urbanization, and accreditation of medical institution. Model 2 was adjusted for length of hospital stay and the covariates in Model 1. Model 3 was adjusted for the number of body parts injured, surgical operations, and the covariates in Model 2. Model 4 was adjusted for transport mode and the covariates in Model 3. Model 5 was adjusted for TMPM and the covariates in Model 4.

Table 4 presents the results of LOS and hospital costs for MVCI patients with and without dementia. The mean LOS for MVCI patients with and without dementia was 11.09 and 8.71 days, respectively, with a significant covariate-adjusted difference in the log means of LOS between the two groups (β = 0.09, 95% CI, 0.04–0.14). Additionally, patients with dementia incurred higher mean hospital costs than did those without dementia (NTD 83,458 vs. NTD 62,338), with a significant covariate-adjusted difference in the log means of inpatient costs between the two groups (β = 0.17, 95% CI, 0.11–0.23).

**Table 4:** Comparison of LOS and inpatient costs between MVCI patients with and without dementia.

Variables	MVCI with dementia		MVCI without dementia		Difference in log (means)					
	Mean	SD	Mean	SD	Crude β (SE)	95%CI	*P*	Adjusted β^†^ (SE)	95%CI	*P*
LOS, days	11.09	21.04	8.71	25.05	0.17(0.02)	0.13–0.22	0.001	0.09(0.02)	0.04–0.14	0.001
Costs, NTD	83,458	135,239	62,338	145,551	0.25(0.03)	0.19–0.31	0.001	0.17(0.03)	0.11–0.23	0.001

*MVCI* motor vehicle crash injury, *LOS* length of hospital stay, *SD* standard deviation, *NTD* New Taiwan Dollars, *β* regression coefficient, *SE* standard error, *CI* confidence interval.

^†^Estimated from the multivariable linear regression model with adjustment for estimated dementia, gender, age, admission year, CCI, urbanization, accreditation of medical institution, number of body parts injured, TMPM, and transport mode.

## Discussion

### Main findings

According to our literature review, this is the first population-based nationwide cohort study to investigate the association of YOD with the hospital outcomes of MVCI inpatients. This study revealed that dementia was associated with an increased risk of ICU admission and respiratory failure in MVCI patients. Although increased in-hospital and 30-day mortalities were also found among patients with an MVCI and comorbid with YOD, the risk of death was not significantly increased in these patients after further controlling for transport mode. Nevertheless, dementia was also positively associated with LOS and hospital costs among MVCI patients.

### Association of dementia with ICU admission and organ failure in hospitalized MVCI patients

Studies have shown that dementia was associated with a higher risk of specific organ failure^32,33^ and could lead to an increased use of intensive care to maintain life^32^. Similar to these studies, our study revealed that dementia was associated with a 63% greater risk of respiratory failure and a 50% higher risk of ICU admission. However, except for respiratory system organ dysfunction, dementia was not associated with the risk of organ dysfunction in other systems. Our findings may be explained by the following. First, our study found that vital visceral organs such as the brain and chest were injured more in the dementia group than in the nondementia group. Among the organ injuries, the proportion of brain injuries differed the most between the two groups. This condition may result in patients with dementia having a higher risk of respiratory failure than patients without dementia because brain injury is mainly associated with an increased risk of respiratory dysfunction^34,35^. Second, YOD was associated with an increased risk of swallowing problems^36^. The swallowing problems from dementia may result in aspiration pneumonia and an attendant increased risk of respiratory failure among patients with dementia^37^. These findings have crucial implications for caring for patients with an MVCI and dementia. Because of the higher risk of respiratory failure among MVCI patients with dementia, airway protection is vital for these patients.

### Association of dementia with in-hospital and 30-day mortality in hospitalized MVCI patients

Studies have suggested that hospitalized patients with dementia have significantly worse prognosis and an increased risk of in-hospital mortality than those without dementia^32,33^. In contrast to these studies, although our study found dementia to be associated with an increased risk of in-hospital and 30-day mortality in MVCI patients, the association was not significant when transport mode was considered. This might indicate that the association of dementia with the risk of in-hospital and 30-day mortality may vary with transport mode. For example, we further found that the association of dementia with the risk of in-hospital mortality was significantly increased in motorcycle crash-related hospitalizations (OR: 1.41, 95% CI, 1.01–1.96) but not in other types of crash-related hospitalizations (data not shown). Why this is so is unclear. Studies have found younger motorcyclist victims to have a greater risk of severe injuries from single-vehicle crashes, which possibly relates to speedy driving^20,38^. Thus, we suspect that MVCI patients with YOD tend to engage in more risk-taking behaviors such as speedy driving when riding a motorcycle^39^ and therefore experience more severe vehicle crashes^40^. This phenomenon thus contributes to the positive association of dementia with the risk of hospital mortality from motorcycle crashes. Additionally, compared with those without, patients with dementia are more often safer road users using simpler conveyances such as a bicycle and less often unsafe road users driving a motor vehicle motorcycle^20^, which may help to reduce the difference in the severity of the injury between the two groups. This would also account for the lack of a significant positive association of dementia with the risk of in-hospital mortality in other nonmotorcycle crash-related hospitalizations. Further studies are warranted to investigate what factors or mechanisms may affect this association.

### LOS and hospital cost in patients with MVCI and dementia

Currently, information regarding the association of dementia with LOS and hospital cost because of MVCI is lacking. A significant result was observed in the present study, namely that patients with MVCI and dementia were more likely to have a longer LOS and higher hospital costs. The severe complications of dementia such as abnormal swallowing function and infection during hospitalization^36^ make MVCI patients susceptible to severe comorbidities such as aspiration pneumonia^37^ and sepsis^32^, which result in a longer LOS and higher hospital costs. A Taiwanese study found that the mean LOS and hospital cost for MVCI patients were 10.7 days and NT$63,242, respectively, which are similar to our findings^41^. Moreover, the LOS and hospital costs for MVCI patients with dementia significantly exceeded those for MVCI patients without dementia in our study. The higher rates of organ failure and ICU admission in the dementia cohort than in the nondementia cohort, which necessitate more life support treatment during hospitalization, may also partially explain these findings.

### Strengths and limitations

This study has some strengths that deserve to be highlighted. First, our study is the first to investigate the association of dementia with the risk of ICU admission, various types of organ failure, and in-hospital and 30-day mortalities; the LOS; and hospital costs in hospitalized patients with MVCI. We used two large, nationwide databases (NHIRD and PTAR), which allowed for the adjustment of many potential confounding factors such as patient demographics, CCI, urbanization level, and hospital level. Second, nearly all medical services were covered by the NHI program in Taiwan. Therefore, the data from the NHIRD used in this study also allowed for the acquisition of complete medical information when measuring the medical utilization and hospital outcomes.

Our study has some limitations. First, the diagnosis of dementia only relied on the coding in the claims data, which might have resulted in disease misclassification and underestimation of dementia in our study. To address this issue, we used at least 2 dementia-related diagnoses with the first and last visits separated by > 90 days, which largely decreased the likelihood of disease misclassification. Second, the treatment for trauma and dementia was improved steadily from 2006 to 2015, thus outcomes of the study population could have affected by the improvement during the ten-year period. To account for the period effect, the year of admission was adjusted in the regression models. However, this study was unable to adjust for the treatment variations among years, which might have led to some degrees of residual confounding bias. Third, because some information was lacking in the claims data, this study could not completely adjust for some important potential confounders such as risky driving behavior and the severity of dementia. However, given that patients with severe dementia are less likely to drive cars, ride motorcycles or bicycles, or walk outside, our dementia cohort likely had a greater proportion of patients with mild-to-moderate-severity dementia. Fourth, this study did not include information on people who immediately died in road accidents. Because non-hospitalization for an MVCI was not included in our study, the conclusions of our analysis were limited to patients hospitalized for an MVCI. Fifth, because the physiologic information of trauma injury such as vital sign and Glasgow Coma Scale is unavailable in our claim data, the analytical models could not be adjusted for the physiological severity such as revised trauma score by Trauma and Injury Severity Score (TRISS) methods. This condition might induce biased results. However, previous studies indicated that TMPM and TRISS have similar mortality predictive abilities^42–44^. Thus, using TMPM as a proxy measure of physiological severity of the trauma injuries should be substantially mitigate the bias of our results.

### Conclusions and implications

In conclusion, this study revealed that among MVCI patients, having dementia is associated with higher risks of ICU admission and respiratory failure, a longer LOS, and higher hospital costs. Additionally, dementia may have a positive association with the increased risk of in-hospital and 30-day mortalities, depending on transport mode. More clinical focus should give to MVCI patients who have comorbid dementia, especially those who are motorcyclists, to further decrease the adverse associations of dementia with MVCI.

## Data availability

The data that support the findings of this study are available from the Ministry of Health and Welfare, R.O.C. Restrictions apply to the availability of these data, which were used under license for this study. Data are available from the Health and Welfare Data Science Center (https://dep.mohw.gov.tw/dos/cp-5119-59201-113.html) with the permission of the Ministry of Health and Welfare, R.O.C.

## Supplementary Information

Supplementary Information.
