# Supplementary material for: Risks of organ failures and deaths associated with young-onset dementia after hospitalizations for motor vehicle crash injuries: a nationwide population-based retrospective cohort study
Source: Sci Rep. 2023 Mar 13;13:4145. doi: 10.1038/s41598-023-30868-6 (PMC10011544; doi:10.1038/s41598-023-30868-6)
Supplement: Supplementary file 1 — Supplementary Information. [file 41598_2023_30868_MOESM1_ESM.docx]

**eTable 1** ICD-9-CM list of the types of injury resulting in MVCI-related hospitalization

| Type of injury | ICD-9-CM |
| --- | --- |
| Fracture | 800-829 |
| Dislocation | 830-839 |
| Sprains and strains | 840-848 |
| Intracranial/ internal injury | 850-869 |
| Open wound | 870-897 |
| Superficial injury/ contusion | 900-924 |
| Other and unspecified effects of external causes | Crushing (925-929)、Foreign body entering through orifice (930-939)、Burn (940-949)、Injury to nerves and spinal cord (950-957)、Poisoning (960-989)、Other injuries (958-959, 990-999) |

**eTable 2** ICD-9-CM list of the body of injury resulting in MVCI-related hospitalization

| Type of injury | ICD-9-CM |
| --- | --- |
| Brain | 850-854 |
| Chest | 860-862 |
| Limbs | 800-848 |
| Abdomen | 863-866, 868 |
| Skin | 870-897, 910-924 |
| Others | 900-909, 925-999 |

**eTable3** ICD-9-CM list of the organ failure

| Organ failure | Codes | Code Description |
| --- | --- | --- |
| Cardiovascular | 458.0 | Hypotension, postural |
|  | 458.8 | Hypotension, specified type, not elsewhere classified |
|  | 458.9 | Hypotension, arterial, constitutional |
|  | 785.5 | Shock |
|  | 785.51 | Shock, cardiogenic |
|  | 785.59 | Shock, circulatory or septic |
|  | 796.3 | Hypotension, transient |
| Respiratory | 518.81 | Acute respiratory failure |
|  | 518.82 | Acute respiratory distress syndrome (ARDS) |
|  | 518.85 | ARDS after shock or trauma |
|  | 786.09 | Respiratory insufficiency |
|  | 799.1 | Respiratory arrest |
| Renal | 584.x | Acute renal failure |
| Hepatic | 570 | Acute hepatic failure or necrosis |
|  | 572.2 | Hepatic encephalopathy |
|  | 573.3 | Hepatitis (septic & not elsewhere classified) |
|  | 573.4 | Hepatic infarction |
| Neurologic | 293 | Transient organic psychosis |
|  | 348.1 | Anoxic brain injury |
|  | 348.3 | Encephalopathy, acute |
|  | 780.01 | Coma |
|  | 780.09 | Altered consciousness, unspecified |
| Hematologic | 286.2 | Disseminated intravascular coagulation |
|  | 286.6 | Purpura fulminans |
|  | 286.9 | Coagulopathy |
|  | 287.3-5 | Thrombocytopenia, primary, secondary or unspecified |
|  | 790.92 | Abnormal coagulation profile |
| Metabolic | 276.2 | Acidosis, metabolic or lactic |
